# Oral candidiasis in Chikungunya viral fever: a case report

**DOI:** 10.1186/1757-1626-3-6

**Published:** 2010-01-05

**Authors:** Jairaj C Kumar, Y Vivek, PK Sudhindra, BD Dhananjaya, Amith T Kumar, Kumar Guru, Arunachalam Kumar, Monappa B Hegde

**Affiliations:** 1 KS Hegde Medical Academy, Nithyananda nagar, Deralakatte, Mangalore 575001, India; 2 Kasturba Medical College, Mangalore, India

## Abstract

A 32 year old Indian male patient presented with chief complaints of a high fever, erythema on ear, severe polyarthritic joint pains & swelling, non pitting pedal oedema, facial puffiness and itching for past four days. He had no significant past medical and drug history and was serologically confirmed to have Chikungunya. Oral cavity inspection revealed whitish non erythematous pseudo membranous plaques on the hard palate, buccal surface of cheek and the floor of the mouth which was later microbiologically confirmed as Candidiasis. He tested negative for HIV and had leucopenia with severe CD4 T-lymphocytopenia. This is the first report of an opportunistic infection with CD4 T-lymphocytopaenia in Chikungunya fever.

## Case Report

In May 2008, a 32 year old Indian male patient presented with chief complaints of a high fever with erythema on ear, severe polyarthritic joint pains & swelling, non pitting pedal oedema, facial puffiness and itching for four days in a Chikungunya viral epidemic outbreak declared region of South Canara district, Karnataka, India. He had no significant past medical and drug history. He had no history of smoking. The patient's BMI was 21.6 kg/m^2^, Blood Pressure was 110/70 mmHg, Pulse rate was 98/min and Respiratory rate was 16/min with no signs of dehydration. The clinical examination of the Respiratory, Cardiovascular, Gastrointestinal and Neurological systems was unremarkable. A provisional clinical diagnosis of Chikungunya was made.

IgM antibody specific to Chikungunya virus was detected using MAC-ELISA. Blood counts revealed leucopenia (2000 cells/micro litre) along with lymphopenia (500cells/Micro litres). The peripheral smear also revealed lymphocytopenia, with the differential lymphocyte Count as 8%, among which 95% were identified as atypical lymphocytes with oval to round condensed nuclear fragments varying in number with toxic granules.

Other laboratory investigations for electrolytes, liver function tests and renal function tests were unremarkable. The ECG of the patient was normal. The patient was put on Paracetamol 500mg thrice daily with Diclofenac 50mg BD and Ranitidine 150mg BD for two days and advised to come for review after two days.

**Figure 1:**
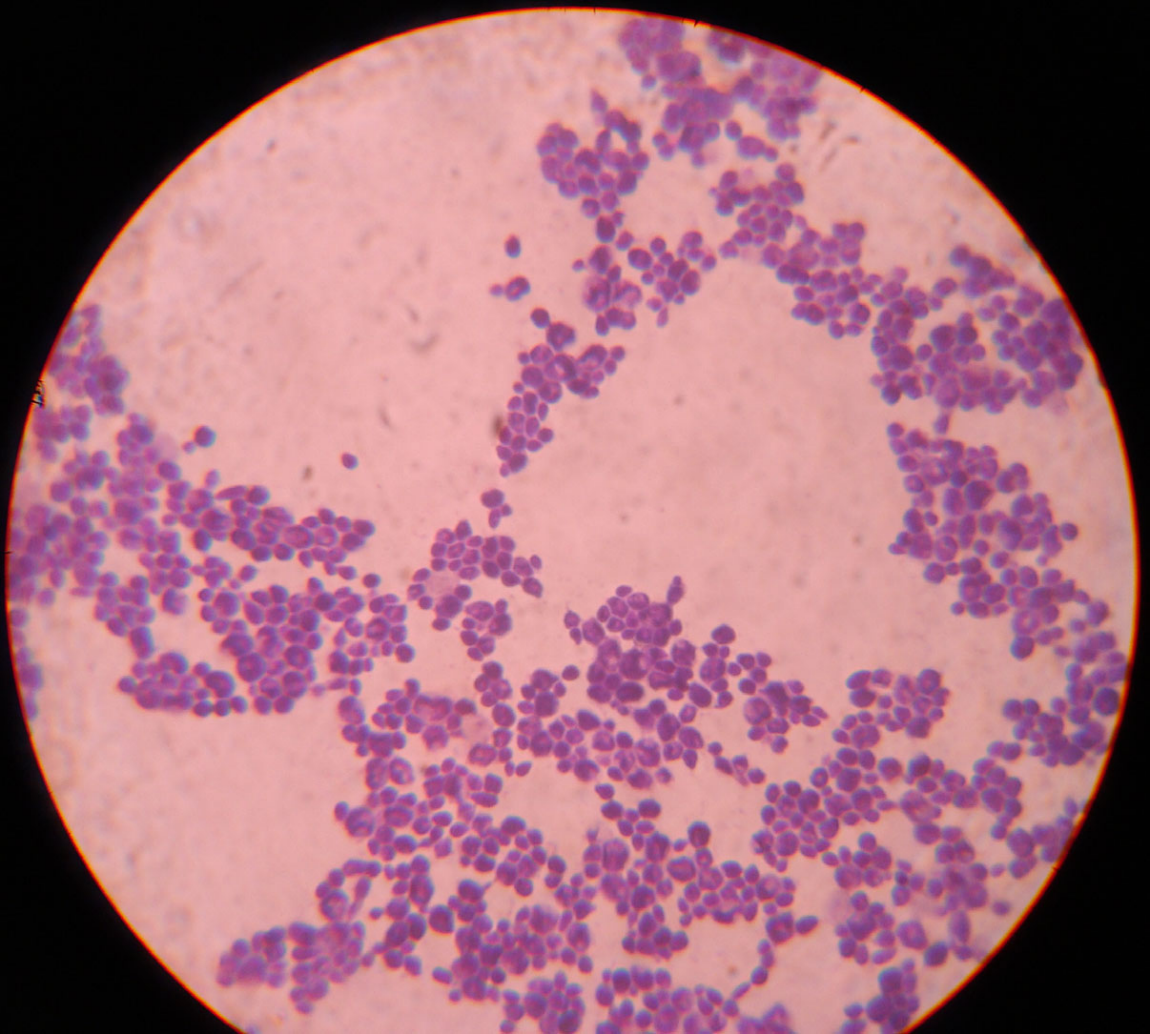
**Oral cavity inspection on review revealed whitish non erythematous pseudo membranous plaques on the hard palate, buccal surface of cheek and the floor of the mouth, later microbiologically confirmed as Candidiasis**.

Oral cavity inspection on review revealed whitish non erythematous pseudo membranous plaques on the hard palate, buccal surface of cheek and the floor of the mouth, later microbiologically confirmed as Candidiasis [figure 1]. After noting the Candidiasis plaques, the patient's blood sample was again sent for CD4 lymphocyte count analysis and HIV conclusive diagnostic tests using ELISA and western blot. The results showed HIV negative and CD4 lymphocytes of 260 cells/micro liter. In addition, we also noted oral Candidiasis in several other Chikungunya patients during this epidemic. Hence, we postulate that the Chikungunya viral fever induced transient immune depression which could lead to the entry of potential Opportunistic Infections such as Candidiasis.

These observations cry out for the better understanding of the impact of Chikungunya viral infection on an already HIV positive individual's susceptibility to opportunistic infections and the progression to dreaded terminal Acquired Immune Deficiency Syndrome (AIDS).

An epidemic of Chikungunya fever of unprecedented magnitude occurred in many parts of India in early 2006 after an interval of 33 years, and there had been resurgence in some parts of South India since June 2007. We have little knowledge of the complications of this debilitating vector borne viral disease. Recent research, on laboratory analysis of 157 Chikungunya patients documented severe lymphopenia and hypocalcaemia as the most prominent findings [1]. Our experience with Chikungunya also showed lymphopenia with a decrease in the CD4 lymphocyte count level. According to the best of our knowledge, we believe that this report is the first report to document the CD4 lymphopenia in Chikungunya with opportunistic Candidiasis infection. We believe that this report shall be of immense importance to all physicians managing immune depressed patients like HIV, Cancer etc.

## Consent

Written informed consent was obtained from the patient for publication of this case report and accompanying images. A copy of the written consent is available for review by the Editor in-Chief of this journal.

## Competing interests

The authors declare that they have no competing interests.

## Authors' contributions

CJK, YV, PKD were responsible for the management of patient and were a major contributors for writing the manuscript. BDD, TAK, performed the microbiological and serological analysis. KG, AK performed peripheral smear examination. BMH had suggested CD4 T-Lymphocyte analysis and also interpreted and analyzed data. All authors have read and approved the final manuscript.
